# Lifestyle coaches as a central professional in the health care network? Dynamic changes over time using a network analysis

**DOI:** 10.1186/s12913-021-06252-3

**Published:** 2021-03-19

**Authors:** Celeste E. van Rinsum, Sanne M. P. L. Gerards, Geert M. Rutten, Ien A. M. van de Goor, Stef P. J. Kremers, Liesbeth Mercken

**Affiliations:** 1 Department of Health Promotion, Maastricht University, P.O. Box 616, Maastricht, 6200 MD The Netherlands; 2 NUTRIM School of Nutrition and Translational Research in Metabolism, Maastricht University, P.O. Box 616, Maastricht, 6200 MD The Netherlands; 3 Faculty of Science and Engineering, University College Venlo, Maastricht University, P.O. Box 8, Venlo, 5900 AA The Netherlands; 4 Department Tranzo, Tilburg School of Social and Behavioral Sciences, Tilburg University, P.O. Box 90153, Tilburg, 5000 LE The Netherlands; 5 CAPHRI Care and Public Health Research Institute, Maastricht University, P.O. Box 616, Maastricht, 6200 MD The Netherlands

**Keywords:** Network analysis, Dynamic changes over time, Lifestyle coaching, Health care professionals, Overweight, Obesity, Combined lifestyle intervention

## Abstract

**Background:**

Overweight and obesity are problems that are increasing globally in both children as well as adults, and may be prevented by adopting a healthier lifestyle. Lifestyle coaches counsel overweight and obese children (and their parents) as well as adults in initiating and maintaining healthier lifestyle behaviours. It is currently unclear whether this novel professional in the Dutch health care system functions as a linchpin in networks that evolve around lifestyle-related health problems. The aim of the present study is to investigate the formation and development of networks of lifestyle coaches and their positions within these networks.

**Methods:**

In this longitudinal study, key professionals and professionals within relevant organisations in the Coaching on Lifestyle (CooL) care networks were asked to fill in three online questionnaires. Respondents were asked to indicate whether they collaborated with each of the specified professionals in the context of CooL. The overall network structures and the central role of the lifestyle coaches were examined by using network analysis.

**Results:**

The results showed that the networks in three out of four regions were relatively centralised, but that none of the networks were dense, and that the professionals seemed to collaborate less with others over time. Half of the lifestyle coaches had a high number of collaborations and a central position within their networks, which also increased over time. In half of the regions, the lifestyle coaches had increased their role as consultants, while their role as gatekeeper and liaison decreased over time. In most regions, the sector of lifestyle coaches had a central position in their networks in just one measurement. Other central sectors were the local sports organisation, public health services, youth health care and the municipal government.

**Conclusions:**

Overall, we cannot conclude that more central and denser networks were formed during the study period. In addition, the lifestyle coaches were not often positioned as a central sector within these networks. Entrepreneurial, network and brokering competences are required for lifestyle coaches to build up denser networks.

**Trial registration:**

NTR6208; date registered: 13–01-2017; retrospectively registered; Netherlands Trial Register.

## Background

Overweight and obesity are problems that are increasing globally in both children as well as adults, and may be prevented by adopting a healthier lifestyle [1, 2]. Due to the complexity of these problems and the variety of causes and consequences [3], many health and non-health professional disciplines are involved in helping to overcome them, and intersectoral collaboration is needed [4]. In addition to these professionals, the lifestyle coach is a novel profession in the Dutch health care system concerning obesity prevention and treatment. Their primary task is being a health care provider, as they counsel overweight and obese children (and their parents) as well as adults in initiating and maintaining healthier lifestyle behaviours. Furthermore, lifestyle coaches are supposed to collaborate with public health and care professionals from whom they receive referrals and to whom they can refer individuals. This referring role is designed to provide an optimal, sustained and tailored treatment for their participants. Thus, lifestyle coaches are likely to function as linchpins (i.e. having a central and connecting role) in the prevention and health care network [5].

In a previous study, it was observed that a coordinating role in a combined lifestyle intervention (CLI) was crucial for a successful collaboration between the professionals and the maintenance of their networks [5, 6]. Lifestyle coaches are considered as new coordinators in networks that evolve around lifestyle-related health problems, and can therefore operate in the centre of CLIs. Since their role is relatively new, however, it has not yet been investigated whether they actually take up this coordinating role in health care networks. Up till now, the networks around lifestyle interventions and lifestyle coaches have not been studied. The aim of the present study is to investigate the formation and development of the networks of lifestyle coaches. By using network analysis, we explored the structure of health care networks and the position of lifestyle coaches within these networks over time. Network analyses have already shown their relevance in studying the health sector [7, 8]. It is expected that the lifestyle coaches will become the central professional regarding obesity in their network over time.

## Methods

### Design and study setting

In the current study, the lifestyle coaches were working in the Coaching on Lifestyle (CooL) intervention, which is a CLI. The protocol of the study on the CooL intervention, details on the intervention [9], the lifestyle changes among CooL participants [10], and the implementation process evaluation [5] are reported elsewhere.

The current longitudinal study examined the position of the lifestyle coaches in the four health care networks they were involved in and how their functioning and the networks’ structure evolved over a two-year period from 2014 to 2016. The lifestyle coaches collaborated with many professionals and organisations. Key professionals (e.g. paramedics) and professionals within relevant organisations (e.g. public health services (PHS) and general practices) were contacted and asked to participate. These professionals, representing themselves or the organisation they worked for, were asked each year to fill in an online questionnaire: at the start of the implementation of the CooL intervention (T0, March 2015 for the adult regions and July 2015 for the child regions), after 11 months (T1, February 2016), and after 22 months (T2, January 2017).

### Dutch health care providers in obesity care

Generally, obesity care for adults in the Netherlands starts at the general practices, because it is the task of the professionals within these practices to diagnose and address overweight and obesity. In addition, they provide basic advice about nutrition and physical activity and, if needed, more detailed guidance on lifestyle changes [11]. Furthermore, Dutch citizens can directly contact dietician and physiotherapist practices concerning obesity problems. If more care is needed, people can be referred to a lifestyle coach or to specialised care (e.g. an internist or a surgeon). In addition, people with obesity can be brought into contact with local sports organisations to join local physical activities or with social worker organisations to increase their participation in society.

The key public health care providers for children are youth health care (YHC) nurses and physicians, who are part of the PHS. This is a unique Dutch system of preventive health care in which YHC professionals monitor children’s health and can signal overweight [12]. If more care is needed and children are obese, they can be referred to lifestyle coaches or paediatricians, mostly in collaboration with a dietician, physiotherapist and psychologist [13].

### CooL intervention

The CooL intervention consisted of three different programmes focusing on different age groups: primary school children (aged 4 to 12 years), adolescents (aged 12 to 18 years), and adults (aged 18 years and older) [9]. The design and implementation of the children’s programmes (the one for the primary school children as well as the one for the adolescents) started later than the adults’ programme due to organisational and practical reasons. Therefore, the baseline questionnaire for the child regions was spread a few months later than for the adult regions. The themes addressed in the CooL intervention are physical activity, dietary and behavioural components, sleep, and stress management. Over a period of 8 to 10 months, lifestyle coaches used group and individual sessions to promote sustainable lifestyle changes among the participants.

The seven lifestyle coaches participating in the study on the CooL intervention had completed a postgraduate training course at the Dutch Academy for Lifestyle and Health (AVLEG). They executed the CooL intervention in two regions for adults (Regions 1 and 2) and in two regions for the children’s programme (Regions 3 and 4) in the southern part of the Netherlands. Two lifestyle coaches worked in both Region 1 and Region 4. This study did not have comparison sites, since its primary focus was on the implementation process [9]. The lifestyle coaches were not instructed to improve the existing networks, nor were they specifically trained by the AVLEG in gaining network skills. At the start of the intervention, three lifestyle coaches were already part of the existing networks within their regions.

In each region a project team was appointed, including the CooL project leader and the lifestyle coaches for that region. In addition, several professionals participated in these project teams: employees from the local ‘health care group’ (i.e. coordinating organisation for primary care providers) or from the PHS, a representative of the local sports organisation and an official from municipal governments.

### CooL regions

The CooL Regions 1 and 2 both consisted of multiple municipalities that included both urban and rural areas. Regions 3 and 4 were both larger cities. The number of citizens in the CooL regions varied from 28,000 to 265,000 in 2014 [14]. Since the CooL intervention was a new intervention, the networks were not yet formed around the lifestyle coaches at the start of the implementation, but the development of these networks started from the beginning of the CooL pilot.

### Recruitment and study population

At each of the three measurements, respondents for this network study were carefully selected by the CooL project teams using the same recruitment methods in each region by means of a purposive and snowball sampling strategy. Per measurement moment, the project team members were asked to identify key professionals and key professional organisations that should be in their CooL care network and who were involved to some extent in the CooL intervention. All key professionals and professionals representing organisations identified to play a role in the CooL care network, such as general practices, YHC professionals and local sport organisations, were asked to fill in the online questionnaires. They were also asked to specify participants in the network that were not yet on the list, to enable the research team to update the list of professionals for the next measurement. These new mentioned professionals were approached in the next measurement moment. This resulted in a long list of professionals that included all possible important professionals, which changed over time. At the three measurements, in total 151, 234 and 230 (T0, T1 and T2, respectively) key professionals and professional organisations were asked to participate in the online survey.

### Study procedure

An email was sent to the identified CooL care network professionals describing the study and providing a link to the online questionnaire. Per measurement, two reminder e-mails were sent. Before starting the questionnaire, the respondents had to grant permission for the confidential use of their data, after which they filled in their job description, organisation and the sector they belong to.

### Measurements

To examine the CooL care networks, all included professionals within a region were shown in a roster, placing professionals within their organisations. Respondents were asked to indicate whether they collaborated with each of the specified professionals in the context of CooL. Collaboration was explicitly defined as: “Collaboration or having contact necessary to keep the CooL intervention running, such as referring to a lifestyle coach or referrals from the lifestyle coaches, within the last three months. It also means to align, to set goals, to take actions, to consult or to exchange information with the person concerned or to give each other advice”. In addition, professionals were asked to indicate the level of intensity of collaboration in terms of strength and frequency (1 = low intensity; 2 = moderate intensity; 3 = high intensity). If the respondents did not collaborate with a professional, they were asked to leave the corresponding answer option blank.

### Building towards a network for the analysis

Where multiple professionals within one general practice responded (e.g. two specialised practice nurses and one general practitioner), the rounded average collaboration intensity was calculated. Where a single professional within a general practice responded, the collaboration was counted as present at the intensity reported by that single professional. This decision was based on the presumption that a single individual within one general practice organisation could be involved in a network connection, and thus establish a collaboration even though others in the organisation may not be involved at all [15]. Where professionals did not fill in the network questionnaire but replied by email to explain that they really were not collaborating with anyone in the CooL care network, they were considered as respondents and all their possible collaborations were interpreted as not present.

All CooL care networks were treated as non-directed networks, which means that either one of the two professionals indicated a collaboration. When there was disagreement among two professionals regarding their mutual collaboration, the rounded average collaboration was calculated and used as collaboration intensity. All responding professionals for which data shows no collaboration with any other professional in the network (isolates) were removed from the network and not taken into account during data analysis.

### Data analysis

First, the overall network structures of the four CooL care networks were examined over time. Three network-level statistics (average degree, density, and centralisation based on degree) were calculated in UCINET 6 [16]. Average degree reflects the mean number of collaborations that professionals have within the network. Density indicates the overall connectivity of the network, expressed as a proportion of actual collaborations relative to the total number of possible collaborations, taking into account the intensity of the collaborations. Centralisation is the extent to which collaborations within a network focus around only one or a few professionals, or whether the network is more decentralised with collaborations spread more evenly among professionals.

To examine specifically the central role of the lifestyle coaches within the CooL care networks over time, multiple individual network measures were calculated (degree, degree centrality, closeness centrality, and betweenness centrality), reflecting the lifestyle coaches’ possible advantaged position within their network. Since comparison within and across years was an important goal of the present study, normalised centrality measures are reported. In the upper part of Table 1, all included individual-level network measures are reported together with a description.

**Table 1:** Definition of the network measures

Lifestyle coaches	
***Centrality*** [17]	
Raw degree	The raw number of collaborations each lifestyle coach has.
Degree centrality	The extent to which lifestyle coaches are collaborating with other professionals within the network.
Closeness centrality	The average distance that lifestyle coaches are removed from all other actors in the network.
Betweenness centrality	Extent to which lifestyle coaches lie on the shortest paths connecting two other actors within the network.
***Brokerage scores*** [20]	
Gatekeeper role X ➔ LSC ➔ LSC	The lifestyle coach controls what information passes into their sector, coming from another sector, e.g. general practices (X).
Representative role LSC ➔ LSC ➔ X	The lifestyle coach controls what information passes out of their own lifestyle coach sector towards another sector, e.g. general practices (X).
Consultant role X ➔ LSC ➔ X	The lifestyle coach links two professionals who are otherwise not directly connected, both from the same outside sector, e.g. general practices (X).
Liaison role X ➔ LSC ➔ Y	The lifestyle coach bridges the gap between two otherwise not directly connected professionals, each within two different outside sectors, e.g. general practices and the health care organisation (X and Y).

*LSC* lifestyle coach

We additionally examined the possible brokerage roles of the lifestyle coaches by running the Gould & Fernandez routine in UCINET 6 [16, 19]. This routine makes it possible to examine how often the lifestyle coaches are a gatekeeper, representative, consultant or liaison between other professionals that belong to specific sectors. Table 1 lists all examined brokerage roles, including a description of each specific role.

**Fig. 1:**
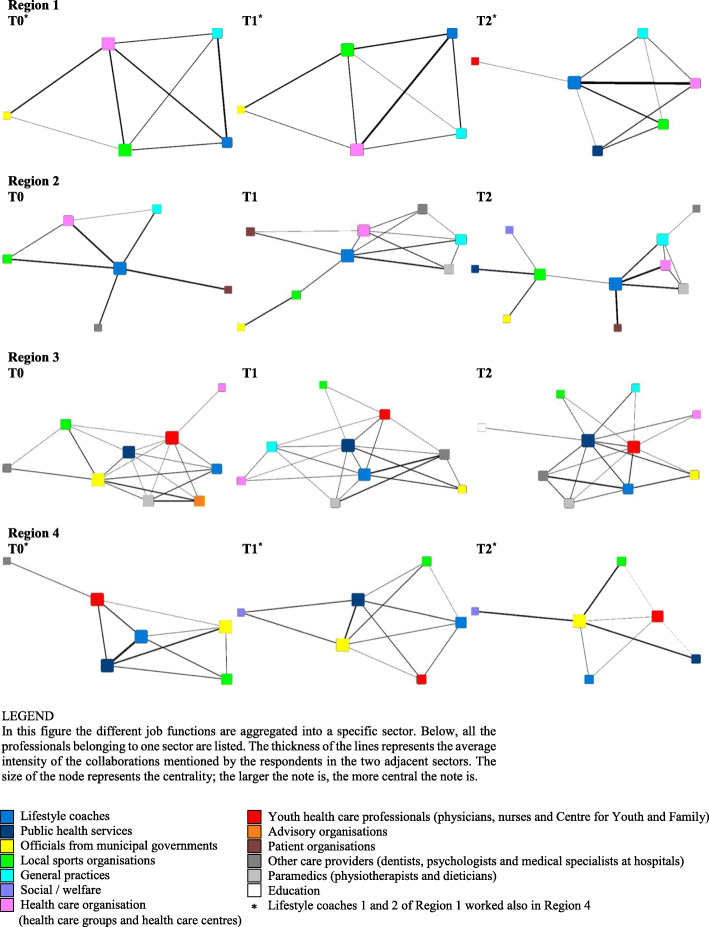
The development of the collaboration between sectors over time per region

Finally, to examine the lifestyle coaches’ collaboration in general within the different sectors, the CooL care networks were aggregated based on the different sectors to which the key professionals and organisations belonged. The aggregated sector networks were visualised in Netdraw, a visualisation routine in UCINET 6 [16]. In the legend of the resulting Fig. 1, the different job functions that were aggregated into a specific sector are presented.

## Results

### Descriptives

Table 2 shows the number of key professionals and professional organisations in the study. In total, 151, 234 and 230 (at T0, T1 and T2, respectively) key professionals and professional organisations were asked to participate in the online survey. On average, 62.6% of the asked network professionals and organisations responded to the questionnaires by indicating whether or not they collaborated with others in the network. The response rates declined over time (i.e. 70.1, 66.9 and 50.9% at T0, T1 and T2, respectively).

**Table 2:** Number of key professionals and professional organisations per region and per measurement

Regions	Measurements	Number of actors^**a**^ asked N (%)	Number of actors^**a**^ responded N (%)	Resulting network size including isolates	Final network size excluding isolates
**Region 1**	**T0**	15	14 (93.3)	15	15
	**T1**	43	34 (79.1)	43	28
	**T2**	53	29 (54.7)	51	32
**Region 2**	**T0**	35	27 (77.1)	35	21
	**T1**	52	43 (82.7)	52	40
	**T2**	58	39 (67.2)	55	44
**Region 3**	**T0**	87	53 (60.9)	85	83
	**T1**	96	50 (52.1)	96	67
	**T2**	88	38 (43.2)	86	73
**Region 4**	**T0**	22	18 (81.8)	22	19
	**T1**	47	33 (70.2)	47	20
	**T2**	33	13 (39.4)	28	14
**Total**^b^	**T0**	157	110 (70.1)	155	136
	**T1**	236	158 (66.9)	236	153
	**T2**	230	117 (50.9)	218	161

^a^Key professionals and professional organisations, ^b^ Two lifestyle coaches, working in two CooL regions, were counted once in this table

### Network descriptive measures over time

To examine the overall network structure of the CooL care networks, several network-level measures were calculated. Table 3 shows all statistics for each region and observation moment separately.

**Table 3:** Network-level measures per region and per measurement moment

Regions	Region 1			Region 2			Region 3			Region 4		
Measurements	T0	T1	T2	T0	T1	T2	T0	T1	T2	T0	T1	T2
**Average degree**	6.133	4.857	3.875	3.714	4.250	3.636	6.289	3.642	4.027	4.632	4.900	2.571
**Density**	0.438	0.180	0.125	0.186	0.109	0.085	0.077	0.055	0.056	0.257	0.258	0.198
**Centralisation**	0.648	0.564	0.624	0.679	0.614	0.618	0.721	0.771	0.956	0.271	0.532	0.397

In general, professionals and professional organisations within the four CooL care networks seemed to collaborate less with others within the network over time (lowering average degree). Density was low within the four CooL care networks, meaning that there were a low number of observed collaborations in relation to the possible number collaborations, and the networks seemed to become slightly sparser over time. When examining the centralisation of the network (i.e. whether the networks centralised around one or several professionals), the regions differ among each other. Regions 1 and 2 were modestly centralised. In Region 4 centralisation was lower and seemed to fluctuate. The CooL care network in Region 3 showed a highly centralised structure over time.

### Centrality of the lifestyle coaches

To examine the central position of the lifestyle coaches in the CooL care networks, the degree and several centrality measures were calculated. Table 4 depicts all these individual measures for each lifestyle coach and observation moment separately.

**Table 4:** Lifestyle coaches’ measures per lifestyle coach and per measurement moment

Region	Lifestyle coach	Measures	T0	T1	T2
Region 1	LSC1^a^	Degree	11	19	22
		Degree centrality	0.786	0.704	0.710
		Closeness centrality	0.824	0.750	0.689
		Betweenness centrality	0.084	0.358	0.505
	LSC2^b^	Degree	12	13	16
		Degree centrality	0.857	0.481	0.516
		Closeness centrality	0.875	0.643	0.646
		Betweenness centrality	0.126	0.120	0.294
	LSC3	Degree			6
		Degree centrality			0.194
		Closeness centrality			0.508
		Betweenness centrality			0.012
	LSC4	Degree			7
		Degree centrality			0.226
		Closeness centrality			0.517
		Betweenness centrality			0.026
Region 2^c^	LSC1	Degree	13	4	
		Degree centrality	0.650	0.103	
		Closeness centrality	0.741	0.481	
		Betweenness centrality	0.287	0.004	
	LSC2	Degree	16	27	29
		Degree centrality	0.800	0.692	0.674
		Closeness centrality	0.833	0.765	0.705
		Betweenness centrality	0.526	0.606	0.779
	LSC3	Degree	9	19	11
		Degree centrality	0.450	0.487	0.256
		Closeness centrality	0.606	0.639	0.500
		Betweenness centrality	0.136	0.189	0.026
Region 3	LSC1	Degree	13	21	24
		Degree centrality	0.159	0.318	0.333
		Closeness centrality	0.539	0.589	0.600
		Betweenness centrality	0.012	0.212	0.042
	LSC2	Degree	17	14	15
		Degree centrality	0.207	0.212	0.208
		Closeness centrality	0.558	0.541	0.558
		Betweenness centrality	0.023	0.038	0.012
Region 4	LSC1^a^	Degree	9	4	1
		Degree centrality	0.500	0.211	0.077
		Closeness centrality	0.643	0.559	0.361
		Betweenness centrality	0.281	0.000	0.000
	LSC2^b^	Degree	3	10	4
		Degree centrality	0.167	0.526	0.308
		Closeness centrality	0.486	0.679	0.542
		Betweenness centrality	0.000	0.082	0.231
Total	Average	Degree	11.444	14.556	13.500
		Degree centrality	0.508	0.415	0.350
		Closeness centrality	0.678	0.627	0.563
		Betweenness centrality	0.164	0.179	0.193
	Standard dev.	Degree	4.187	7.764	9.324
		Degree centrality	0.282	0.216	0.213
		Closeness centrality	0.144	0.095	0.103
		Betweenness centrality	0.173	0.197	0.265

*LSC* lifestyle coach

^a +b^ Both these two lifestyle coaches worked in Region 1 and Region 4, ^c^ LSC1 stopped participating in the CooL pilot and LSC3 took over the role

#### Region 1

In Region 1, four lifestyle coaches were active during our study, although Lifestyle coaches 3 and 4 only joined at the last measurement, showing low degree and centrality at their start-up. Lifestyle coaches 1 and 2 were actively involved from the start of the study and increased their collaboration with other professionals and professional organisations over time. At the first measurement, they were both highly central regarding the number of direct connections (degree centrality) and the average number of steps the lifestyle coaches were removed from everyone in the network (closeness centrality). Over time, however, both lifestyle coaches became less central in this respect. Betweenness centrality was low at the first measurement, but increased over time, especially for Lifestyle coach 1. This implies that over time the lifestyle coaches increased their position of being on the shortest path between two other professionals.

#### Region 2

The three lifestyle coaches from this region all show vastly different centrality patterns. Lifestyle coach 1 showed relatively high degree and closeness centrality, and moderate betweenness centrality at the first measurement. At the second measurement, this coach’s centrality as well as the degree rates decreased strongly. After the second measurement, this lifestyle coach terminated participation as a lifestyle coach in the CooL study. Lifestyle coach 2 had the most collaborations of all lifestyle coaches in the pilot and the highest rates of all centrality measures. Although the degree and closeness centrality showed a minor decrease over time, betweenness centrality increased drastically. This showed that this lifestyle coach maintained a strong central position in the network over time. The scores of Lifestyle coach 3 were compared to the averages of the other lifestyle coaches in the pilot.

#### Region 3

The two lifestyle coaches in this region both showed average closeness centrality, low degree centrality, and very low to none betweenness centrality over the three measurements. Lifestyle coach 1 did show an increase in the number of collaborations over time, whereas Lifestyle coach 2 showed fewer collaborations.

#### Region 4

In Region 4, compared to the other regions, both lifestyle coaches had a low number of collaborations. Both lifestyle coaches showed an average closeness centrality (i.e. an indication of how fast they could reach all other actors within the CooL care network). However, their degree and betweenness centrality over time became very low, which made them badly connected and almost never on the shortest path between two other professionals.

### The brokerage roles of the lifestyle coaches

To examine the brokerage roles of the CooL lifestyle coaches, all actors in the network were assigned to one of the thirteen identified sectors. Table 5 shows how often each lifestyle coach was identified to be a gatekeeper, representative, consultant or liaison. Both raw and normalised counts taking network size into account are reported.

**Table 5:**
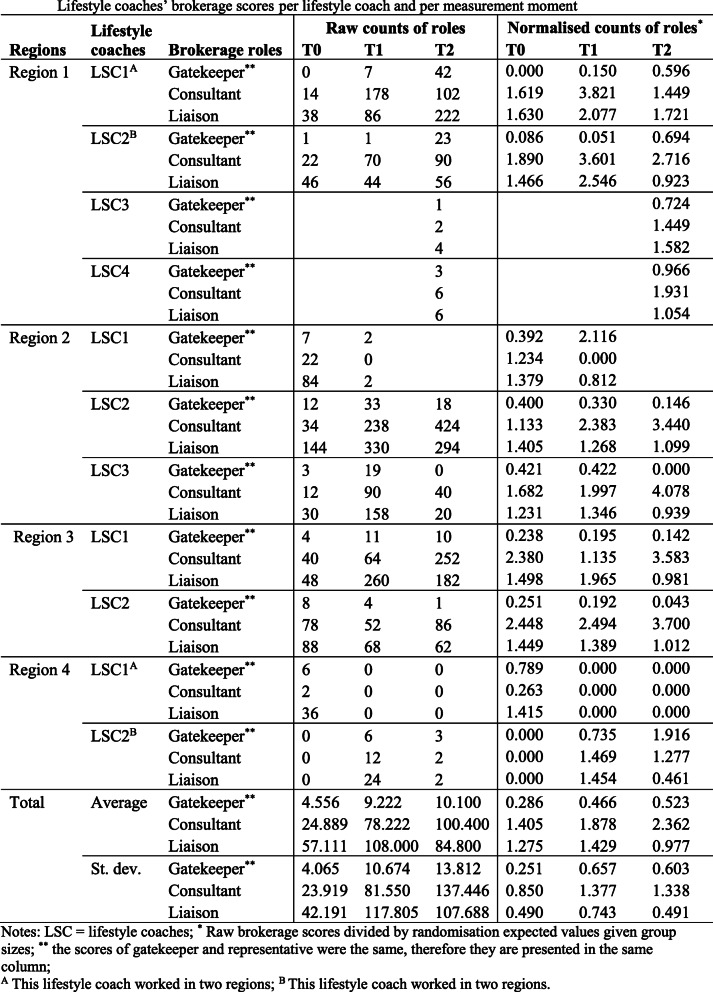
Lifestyle coaches’ brokerage scores per lifestyle coach and per measurement moment

#### Region 1

Lifestyle coaches in Region 1 did not often act as a gatekeeper or representative for their own lifestyle coach sector, although a slight increase over time was noticeable. When being a broker, most of the time they were a broker between two otherwise unconnected professionals working within the same sector (mainly in general practices), followed by being in between two professionals from different sectors (between general practices, local sports organisations and the health care group, and later also YHC professionals and PHS). Especially at T1, their number of connections increased in these two brokerage roles. The raw counts of Lifestyle coach 1’s liaison role even increased steeply at T2.

#### Region 2

In the second region, relative brokerage scores showed that Lifestyle coach 1 was increasingly positioned as a broker between other lifestyle coaches and professionals from other sectors. However, this coach was less often a consultant or a liaison linking other professionals over time, before ending participation as a lifestyle coach in the CooL study. The raw counts show that the role of Lifestyle coach 1 as a broker disappeared at T1. The remaining two lifestyle coaches had the majority of their connections at T1. They both strongly increased their role as consultant (primarily in general practices), while their role in connecting two professionals from different sectors decreased slightly over time (they mainly connected general practices with a variety of other sectors).

#### Region 3

In Region 3, a similar pattern compared to Region 2 can be seen. Both the lifestyle coaches in this region strongly increased their consultancy role (mainly for YHC professionals), while decreasing their liaison role (they mainly connected sectors with YHC professionals and the ‘other care providers’ sector). Their role as gatekeeper or representative diminished over time (they primarily connected the YHC professionals with their own sector).

#### Region 4

The fourth region showed very low involvement in brokerage roles by both lifestyle coaches. Compared to the other CooL care regions, the lifestyle coaches in Region 4 showed the highest tendency to take on a gatekeeper and representative role (they primarily connected YHC professionals and local sport organisations with their colleague lifestyle coach). However, the raw counts indicate that the lifestyle coaches had hardly any connections.

### Aggregated sectoral networks

To examine the lifestyle coaches’ collaboration with different sectors specifically, the CooL care networks were aggregated based on the 13 identified sectors key to which professionals and organisations belonged. Figure 1 shows for each region at each measurement time a visualisation of the sector network. The ideal situation is that the lifestyle coaches would have as many connections as possible and they are central in their network. In addition, the other sectors in the network would make new connections with each other, thanks to the brokering role of the lifestyle coaches. This would make it a strong and dense network with the lifestyle coach as central sector.

The position of the lifestyle coaches in the four CooL regions during the study period can be seen in the visualisations. Especially in Region 2, the lifestyle coaches gained a crucial place within the network over time. At T1, their position became more central. Then at T2, they occupied a central position together with the local sports organisation, where they both connected the two different parts of the network. The local sports organisation was therefore important for the lifestyle coaches in order to reach the other three sectors.

In Region 3, the lifestyle coaches also fulfilled a quite central role in their network at T1. However, at the start the PHS had this central position and at T2 a star figure had been formed with the PHS and YHC as central organisations. In Regions 1 and 4, the lifestyle coaches had a relatively central position in their networks at only one moment (at T2 and at T0, respectively). In Region 4, at T1 the PHS together with the municipal government seemed to be more central, and at T2 the municipal government was the only central sector.

Furthermore, different sectors appeared and disappeared over time in the four CooL regions. The role of the government in Region 1 disappeared at T2. Also at T2, the YHC and PHS appeared in the network, because the network professionals wanted to implement the children’s programme within the region since by then the adults’ programme was well implemented in that region. In Region 2, a dietician entered the network at T1 and immediately had good collaborations with several professionals. In addition, the education sector appeared at T2 in Region 3. This sector consisted of primary school teachers with a special task of physical activity. Additionally, the general practices started in the network at T1. At T1, dentists were included as new collaborators in the network (categorised as ‘other care providers’). Furthermore, in Region 4 the social sector made its entry at T1, because members of the original network started collaborating with these professionals.

Of the four CooL regions, Region 2 approached the ideal situation, due to the central position of the lifestyle coaches. However, in this network the number of connections between sectors fluctuated over time. Moreover, the network depends highly on the lifestyle coaches’ role. If the lifestyle coaches were to disappear, half of the network would have no contact with the other half. It would be better if the lifestyle coaches brought different sectors in contact with each other, so that they knew how to find each other, thereby making the connections sustainable.

## Discussion

The aim of the present study was to investigate the formation and development of networks of lifestyle coaches during the study period. Since their role is relatively new, to our knowledge the development of health care networks around lifestyle coaches and the coaches’ coordinating role in these networks have not previously been studied. Since the Coaching on Lifestyle (CooL) intervention is a new intervention, the networks had not yet been formed around the lifestyle coaches. Because it is assumed that lifestyle coaches may be the linchpin in obesity care networks, it was expected that the lifestyle coaches would evolve towards a more central position in their networks during the study pilot.

The CooL intervention was executed by lifestyle coaches in two regions for adults (Regions 1 and 2) and in two regions for children (Regions 3 and 4). In the network of Region 1, the organisations collaborated less with others and the network was modestly centralised and not dense. The lifestyle coaches achieved more collaborations and a more central position over time. They were increasingly positioned as liaison brokers. The sector of the lifestyle coaches occupied a relatively central position in the network only at T2. That the professionals collaborate less with each other over time can be explained by the fact that the CooL pilot started with a small number of referrers in this region. This was later expanded to more referrers from which to receive more referrals. The first group of referrers remained active collaborators in the network. Furthermore, professionals in this region wanted to implement the children’s programme, which led to incorporation of new sectors.

In the network of Region 2, the organisations collaborated less with others and the network was modestly centralised and not dense. One of the lifestyle coaches had a high number of collaborations and had a central position. The lifestyle coaches strongly increased their role as consultants and their role as gatekeeper and liaison decreased slightly. The sector of the lifestyle coaches acquired a crucial place within the network over time, but later they occupied a central position together with the local sports organisation.

In the network of Region 3, the organisations collaborated less with others and the network was highly centralised and not dense. One of the lifestyle coaches gained more and closer collaborations with other professionals over time. The lifestyle coaches strongly increased their role as consultants and their role as gatekeeper and liaison decreased. The sector of the lifestyle coaches also fulfilled a quite central role in the network. However, at the start, the PHS occupied this central position and at T2 a star figure had been formed with the PHS and YHC as central organisations. Due to its central network and the role of the lifestyle coaches, this region came closest to the expected development.

In the network of Region 4, the organisations collaborated less with others and the network was low centralised and not dense. The lifestyle coaches were not active and their number of collaborations decreased over time. They had very low involvement in the brokerage roles. The sector of the lifestyle coaches only had a relatively central position in the network at T0. At T1, the PHS together with the municipal government seemed to be more central and at T2 the municipal government was the only central sector. These results can be explained by the fact that the lifestyle coaches were not active and did not invest in their network.

The observations during the study period [5] and the results of this network analysis are in line with each other. The central lifestyle coaches with a brokering role performed more entrepreneurial activities, which indicates that these activities are key for playing a central role in the network. In addition, some lifestyle coaches were less present in the network due to their personal situation or work situation.

Lifestyle coaches are seen as linchpins (i.e. having a central and connecting role) in the prevention and health care networks [17]. Fulfilling their linchpin role demanded entrepreneurial activities, and networking and brokering skills. However, it was observed in this study that the lifestyle coaches took up and developed this central role to a lesser degree than expected. A previous study showed that the CooL lifestyle coaches evaluated entrepreneurship as their least strong competence, while they also rated it the least important competence to have as a lifestyle coach [5]. This may partly be due to the fact that the lifestyle coach is a relatively new profession in the Netherlands and that training programmes focussed on coaching skills and did not take into account these more entrepreneurial competences. Only a few lifestyle coaches in the CooL pilot had a natural aptitude for this competence. This was observed in a previous study [17], but it was also evident in this study. Today, most training programmes for lifestyle coaches have integrated entrepreneurship into their programmes. A new study should investigate whether lifestyle coaches currently have more entrepreneurial, networking and brokering skills.

Building up and maintaining a dense network requires entrepreneurial, network and brokering skills, which include taking risks, looking for new opportunities, starting and maintaining relationships, connecting professionals, and combining knowledge [18, 20]. Research has also demonstrated that brokering professionals can use a personal approach to create a shared interest and build trust [21, 22]. Trust among stakeholders has been shown to be essential for building sustainable relationships [21–24]. Where collaborations already exist, trust is more likely to be built up. Therefore, relationships are better and more trust is built up when a professional has a more central position in a dense network [20, 25, 26]. However, building trust takes time and takes place throughout the collaboration process [27].

Another interesting observation in this study is that the central position was shared with other sectors (i.e. local sports organisation, PHS, YHC and the municipal government). From observations in the regions, we assumed that the other sectors had their own connectors and that these connectors knew where to find each other. This can become an ideal situation, in which the professional with the most entrepreneurial competences in their sector is the connector and that these connectors form a strong connection with each other. In this way, intersectoral collaboration can be increased and led by enthusiastic connectors.

### Strengths and limitations

This is the first network analysis of the development of health care networks around lifestyle coaches and the coaches’ coordinating role in these networks. The strengths of this study include its longitudinal design and the comparison between different regions. Furthermore, this study started at the beginning of the intervention’s implementation phase, resulting in a good overview of how the networks developed over time when implementing an intervention.

Being the first to perform this kind of research is also a limitation due to a missing frame of reference. To draw the story per region, we advise further research to combine quantitative network data with qualitative data from the perspective of the key professionals, in this case the lifestyle coaches, and from other implementation factors (e.g. fidelity and reach). In addition, the definition of collaboration could be more specified to gain insights in different type of relationships, for example referring participants or coordinating care. In this study we used a broad definition of collaboration since many different sectors were participating in the CooL intervention. Another limitation was having a lot of missing values. On average, 37.4% of the professionals did not fill in the questionnaire per measurement per region. To reduce the number of missing values, we assumed that there was a mutual collaboration if either one of the two professionals indicated collaborating with the other. This method can be used for up to 40% missing data [28]. The low response rates could have biased the results as connected professionals are more willing to filled in the questionnaires. We assumed that professionals, who did not fill in the questionnaires, were not active participating in the regions as we had wide boundaries for the networks. Since the inactive professionals would have fallen out during the analysis, this would not have a significant impact on the results. Furthermore, since the child regions started implementation at a later stage, the period between baseline and T1 measurement differed for the adult and child regions. This allowed lifestyle coaches in child regions less time to build up their network. This can also lead to biased results as the lifestyle coaches in the adult region had a longer period to build up their networks, but we took this into account when we interpreted the results. This situation is typical when implementing an intervention. Finally, we studied four single regions within a pilot, therefore we cannot generalize these findings to other networks or situations. Despite these limitations, the study reveals initial insights in how networks around lifestyle coaches in obesity care networks develop.

## Conclusions

Overall, we cannot conclude that more central and denser networks were formed during the study period. In addition, the lifestyle coaches were not often positioned as a central sector within these networks. Entrepreneurial, network and brokering competences are required for lifestyle coaches to build up denser networks.

## Data Availability

The datasets used and analysed during the current study are available from the corresponding author upon reasonable request.

## Abbreviations

AVLEG: Dutch Academy for Lifestyle and Health
CLI: Combined lifestyle interventions
CooL: Coaching on Lifestyle
PHS: Public health services
YHC: Youth health care
